# Chitinase 3-like 1 Regulates Cellular and Tissue Responses via IL-13 Receptor α2

**DOI:** 10.1016/j.celrep.2013.07.032

**Published:** 2013-08-22

**Authors:** Chuan Hua He, Chun Geun Lee, Charles S. Dela Cruz, Chang-Min Lee, Yang Zhou, Farida Ahangari, Bing Ma, Erica L. Herzog, Stephen A. Rosenberg, Yue Li, Adel M. Nour, Chirag R. Parikh, Insa Schmidt, Yorgo Modis, Lloyd Cantley, Jack A. Elias

**Affiliations:** 1 Section of Pulmonary, Critical Care and Sleep Medicine, Department of Internal Medicine, Yale University School of Medicine, 300 Cedar Street, New Haven, CT 06520-8057, USA; 2 Molecular Biophysics and Biochemistry Department, Yale University, 266 Whitney Avenue, P.O. Box 208114, New Haven, CT 06520-8114, USA; 3 Section of Nephrology, Department of Internal Medicine, Yale University School of Medicine, 300 Cedar Street, New Haven, CT 06520-8057, USA; 4 Hanover Medical School, 30625 Hanover, Germany

## Abstract

Members of the 18 glycosyl hydrolase (GH 18) gene family have been conserved over species and time and are dysregulated in inflammatory, infectious, remodeling, and neoplastic disorders. This is particularly striking for the prototypic chitinase-like protein chitinase 3-like 1 (Chi3l1), which plays a critical role in antipathogen responses where it augments bacterial killing while stimulating disease tolerance by controlling cell death, inflammation, and remodeling. However, receptors that mediate the effects of GH 18 moieties have not been defined. Here, we demonstrate that Chi3l1 binds to interleukin-13 receptor α2 (IL-13Rα2) and that Chi3l1, IL-13Rα2, and IL-13 are in a multimeric complex. We also demonstrate that Chi3l1 activates macrophage mitogen-activated protein kinase, protein kinase B/AKT, and Wnt/β-catenin signaling and regulates oxidant injury, apoptosis, pyroptosis, inflammasome activation, antibacterial responses, melanoma metastasis, and TGF-β1 production via IL-13Rα2-dependent mechanisms. Thus, IL-13Rα2 is a GH 18 receptor that plays a critical role in Chi3l1 effector responses.

## INTRODUCTION

The 18 glycosyl hydrolase (GH 18) gene family contains true chitinases (Cs) that degrade chitin polysaccharides and chitinase-like proteins (CLPs) that bind to but do not degrade chitin (Lee et al., 2011). They are members of an ancient gene family that exists in species as diverse as plants and humans and has evolved during speciation, with a particularly impressive increase in CLPs coinciding with the appearance of mammals (Aerts et al., 2008; Funkhouser and Aronson, 2007). This retention over species and evolutionary time has led to the belief that these moieties play essential roles in biology. Recent studies have confirmed this speculation (Dela Cruz et al., 2012; Lee et al., 2009, 2011; Lee and Elias, 2010; Sohn et al., 2010). This is particularly true for the prototypic CLP chitinase 3-like-1 (Chi3l1, also called YKL-40 in humans and BRP-39 in mice), which has been shown by our laboratory and others to play major roles in antipathogen, antigen-induced, oxidant-induced, inflammation, repair and remodeling responses by regulating a variety of essential biologic processes including oxidant injury apoptosis, pyroptosis, inflammasome activation, Th1/Th2 inflammatory balance, M2 macrophage differentiation, transforming growth factor β1 (TGF-β1) elaboration, dendritic cell accumulation and activation, and mitogen-activated protein kinase (MAPK) and Akt signaling (Areshkov et al., 2012; Chen et al., 2011a; Dela Cruz et al., 2012; Kim et al., 2012; Lee et al., 2009; Sohn et al., 2010). The potential importance of YKL-40/Chi3l1/BRP-39-induced responses can also be seen in the large number of diseases in which Chi3l1/YKL-40 excess has been documented and the observation that the degree of Chi3l1/YKL-40 dysregulation often correlates with the severity and natural history of these disorders (reviewed in Coffman, 2008; Lee et al., 2011). Surprisingly, the mechanisms via which the GH 18 moieties mediate their biologic effects are poorly understood. Importantly the possibility that GH 18 proteins mediate their biologic effects via a ligand-receptor paradigm has not been addressed, and moieties that bind to and signal in response to any of these regulators have not been defined.

To address the possibility that YKL-40/Chi3l1/BRP-39, which does not have known enzymatic activity, mediates its effects via identifiable receptors, we used yeast two-hybrid binding and colocalization assays to define YKL-40/Chi3l1/BRP-39 binding-partner interactions and assessments of signaling, gene expression, and in vivo phenotype generation to evaluate the consequences of these interactions. These studies demonstrate that YKL-40/Chi3l1/BRP-39 binds to interleukin-13 receptor α2 (IL-13Rα2). They also demonstrate that YKL-40/Chi3l1/BRP-39, IL-13Rα2, and IL-13 are in a multimeric complex. Lastly, they demonstrate that YKL-40 activates MAPK, Akt, and Wnt/β-catenin signaling pathways and regulates apoptosis, pyroptosis, inflammasome activation, oxidant injury, antibacterial responses, melanoma metastasis, and TGF-β1 elaboration via IL-13Rα2-dependent mechanisms.

## RESULTS

### Chi3l1/YKL-40/BRP-39 Binding to IL-13Rα2

**Figure 1:**
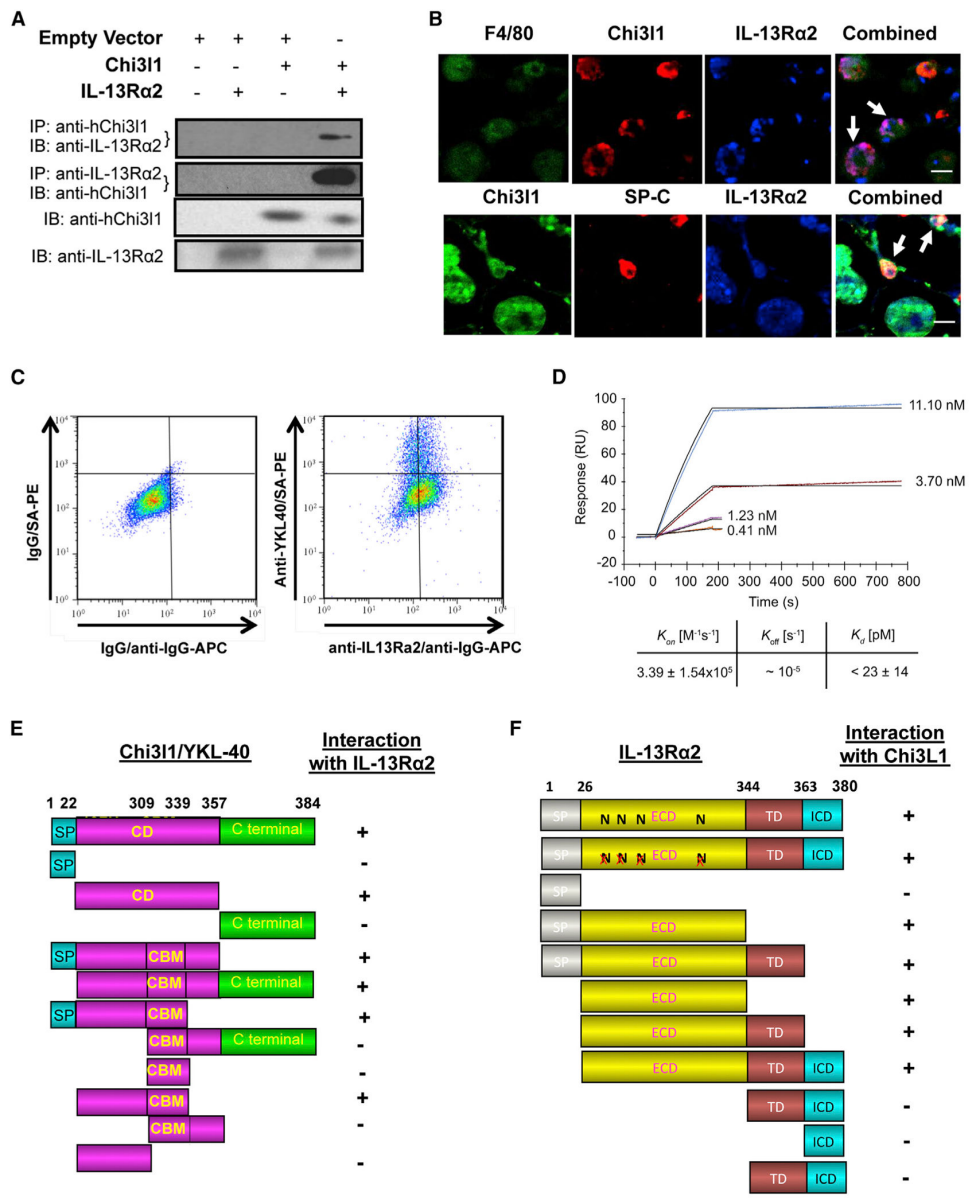
Binding and Localization of Chi3l1/YKL-40 and IL-13Rα2 (A) A549 cells were transfected with Chi3l1/YKL-40 (Chi3l1) and/or human IL-13Rα2 (IL-13Rα2), lysates were prepared and immunoprecipitated (IP) with either anti-Chi3l1 or anti-iL-13Rα2, and the precipitates were evaluated using immunoblot (IB) analysis as noted. (B) Triple-label IHC to detect the colocalization of IL-13Rα2 and BRP-39 in the macrophages (upper panels) and type 2 alveolar epithelial cells (lower panels) in lungs from IL-13 Tg mice using antibodies to BRP-39, IL-13Rα2, and cell-specific markers of macrophages (anti-F4/80) and type 2 epithelial cells (anti-SP-C). Arrows highlight some of the colocalized cells. (C) Cell surface colocalization of Chi3l1/YKL-40 (Chi3l1) and IL-13Rα2 (IL-13Rα2). THP-1 cells were incubated in the presence or absence of anti-YKL-40-biotin antibody and anti-iL-13Rα2 immunoglobulin G (IgG) antibody without permeabilization. They were then washed and stained with streptavidin (SA)-PE and anti-igG-APC and subjected to flow cytometric analysis. (D) Measurement of the affinity and kinetics of IL-13Rα2 binding to Chi3l1/YKL-40 by surface plasmon resonance (SPR). Chil3l1/YKL-40 was immobilized and IL-13Rα2 was in the mobile phase. (E) Yeast two-hybrid characterization of the structures in hChi31 that bind to IL-13Rα2. The 18 GH catalytic domain (CD), chitin binding motif (CBM), signal peptide (SP), and C-terminal fragment are illustrated. (F) Yeast two-hybrid characterization of the structures in IL-13Rα2 that bind to hChi31. The extracellular domain (ECD), transmembrane domain (TD), intracellular domain (ICD), signal peptide (SP), and sites of N-glycosylation (N) are illustrated. Each panel is representative of a minimum of three evaluations. Scale bars in (B) represent 10 μm. See also Figure S1.

To define the binding partners of Chi3l1/YKL-40, yeast two-hybrid analysis was undertaken using Chi3l1/YKL-40 as bait. A number of clones gave positive results in these assays. One of the most intriguing encoded IL-13Rα2 (Figure S1A). Further documentation of the interaction between YKL-40 and IL-13Rα2 was obtained with coimmunoprecipitation (coIP), colocalization, and Biacore assays. In the former, A549 cells were transfected with both of these moieties and subjected to immunoprecipitation (IP) with antibodies to one moiety, and the precipitate was then analyzed via western blotting using antibodies to the other moiety. In these experiments, the two moieties always traveled together with IP using antibodies against YKL-40 always precipitating IL-13Rα2 and vice versa (Figure 1A). Immunohistochemical evaluations of lungs from IL-13 transgenic (Tg) mice (in which Chi3l1/BRP-39 and IL-13Rα2 are both strongly induced) demonstrated that Chi3l1/BRP-39 and IL-13Rα2 frequently colocalize in these tissues (Figure 1B). The major site of this colocalization was in F4/80+ macrophages (Figures 1B and S1B). Interestingly, there were macrophage populations in which colocalization occurred and populations in which Chi3l1 was noted and IL-13Rα2 could not be detected (Figure 1B). Colocalization in some alveolar type II cells was also appreciated (Figures 1B and S1C). To identify the sites in the cell of this colocalization, we employed fluorescence-activated cell sorting (FACS) evaluations of nonpermeabilized cells and immunohistochemistry (IHC) of tissue sections and stained both with anti-Chi3l1 and anti-iL-13Rα2. These studies clearly demonstrate that Chi3l1 and IL-13Rα2 can be seen together on the surface and in the cytoplasm of the cell (Figures 1C and S1D). The Biacore assays also demonstrated that Chi3l1/YKL-40 and IL-13Rα2 bind to one another. At pH 7.4, the binding was quite avid, with a *K_d_* of 23 ± 14 pM (Figure 1D). The *k_off_* was approximately 10^−5^ s^−1^ and the *k_on_* was 3.39 ± 1.54 × 10^5^ M^−1^ s^−1^. These studies demonstrate that YKL-40 specifically binds to IL-13Rα2 with high affinity.

### Localization of Chi3l1/YKL-40-IL-13Rα2 Binding

Deletion mapping was next employed to define the regions in Chi3l1/YKL-40 and IL-13Rα2 that are required for their interactions in the yeast two-hybrid assay. These studies demonstrated that the region of YKL-40 between amino acids 22 and 357 contained the elements that were required to bind to full-length IL-13Rα2 (Figure 1E). This region is called the catalytic domain (CD) of GH 18 moieties and contains the chitin binding motif (CBM) but does not contain its signal peptide (SP) or the C-terminal peptide (Figure 1E). Interestingly, the CBM was necessary, but not sufficient, for YKL-30/Chi3l1-IL-13Rα2 binding (Figure 1E). These studies also demonstrated that the extracellular domain (ECD) of YKL-40 contained elements that were required for binding to full-length Chi3l1/YKL-40 (Figure 1E). The transmembrane and intracellular motifs of IL-13Rα2 did not play a critical role in this interaction (Figure 1F). The four sites of N-linked glycosylation within the ECD were also able to be mutated without abrogating IL-13Rα2 ECD-Chi3l1/YKL-40 binding (Figure 1F). These studies demonstrate that Chi3l1/YKL-40/IL-13Rα2 binding is dependent on the CD and the CBM of the former and the ECD, but not the sites of N-glycosylation in the latter.

### Interactions with Soluble IL-13Rα2

An ECD-containing soluble version of this receptor has been described elsewhere (Chen et al., 2008, 2009). We thus speculated that Chi3l1/YKL-40 would also bind to soluble IL-13Rα2 (sIL-13Rα2). To test this hypothesis, we did coIP experiments using bronchoalveolar lavage (BAL) fluids from IL-13 Tg mice. These studies demonstrated that Chi3l1/BRP-39 also binds to sIL-13Rα2 (Figure S1E).

### Role of IL-13Rα2 in Chi3l1/YKL-40/BRP-39 Signaling

**Figure 2:**
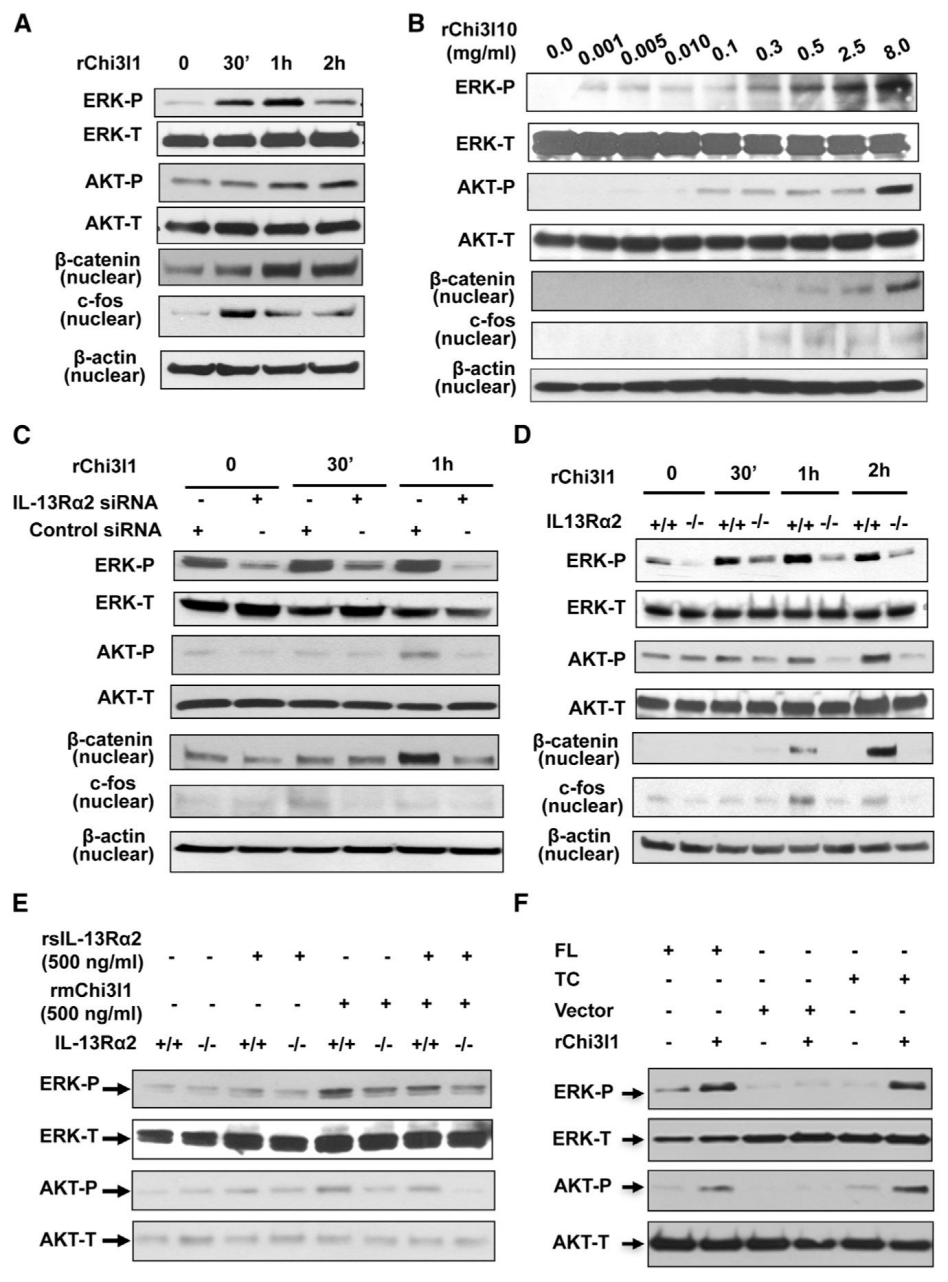
Chi3l1 and IL-13Rα2 Regulation of Macrophage Signaling (A and B) THP-1 cells were incubated with recombinant Chi3l1/YKL-40 (rChi3l1) for the noted periods of time at the noted doses. Western blot evaluations were used to evaluate ERK1/2 phosphorylation (ERK-P), total ERK 1/2 (ERK-T), AKT phosphorylation (AKT-P), total AKT (AKT-T), β-catenin nuclear translocation, and nuclear c-*fos* accumulation. (C and D) The role(s) of IL-13Rα2 in these responses was assessed by comparing these signaling events in THP-1 cells treated with IL-13Rα2 siRNA (siRNA^+^) or scrambled controls (siRNA^−^) (C) and peritoneal macrophages from wild-type (+/+) and IL-13Rα2 null (−/−) mice (D). Each panel is representative of a minimum of three evaluations. (E) The effects of recombinant soluble IL-13Rα2 (rsIL-13Rα2) on Chi3l1-stimulated signaling were also assessed. In these experiments, peritoneal macrophages from WT and IL-13Rα2 null mice were treated with PBS or rsIL-13Rα2 and rChi3l1 as indicated and ERK and AKT activation was evaluated by western blot analysis. (F) Peritoneal macrophages from IL-13Rα2 null (−/−) mice was transfected with full-length (FL) IL-13Rα2, a truncated construct (TC) of IL-13Rα2 that lacked its intracellular domain, or an empty vector. After incubation of the cells with rChi3l1 (500 ng/ml) as indicated, cell lysates were prepared and ERK and AKT activation was evaluated by western blot analysis. See also Figure S2.

To define the role(s) of IL-13Rα2 in Chi3l1/YKL-40/BRP-39-induced intracellular signaling, we compared the effects of Chi3l1/YKL-40/BRP-39 on MAPK, extracellular signal-regulated protein kinase (ERK) 1/2, AKT, and Wnt/β-catenin activation in human THP-1 cells treated with IL-13Rα2 small interfering RNA (siRNA) or scrambled controls and peritoneal macrophages from wild-type (WT) and IL-13Rα2 null mice. As can be seen in Figure 2A, ERK activation, AKT activation, and the induction of nuclear β-catenin and c-*fos* were seen in THP-1 cells 30 min to 2 hr after the addition of recombinant (r) Chi3l1/YKL-40. These effects were dose dependent, with the activation of ERK and AKT being seen with doses of rYKL-40 as low as 0.1–0.3 μg/ml and the induction of nuclear β-catenin and c-*fos* being seen with doses as low as 0.3–0.5 μg/ml (Figure 2B). They were also at least partially IL-13Rα2 dependent, because siRNA that decreased the levels of IL-13Rα2 messenger RNA (mRNA) by greater than 70% (Figures S2A and S2B) significantly decreased each of these activation events (Figure 2C). In accord with these findings, rChi3l1/BRP-39 activated ERK and AKT signaling and induced nuclear β-catenin and c-*fos* accumulation in peritoneal macrophages from WT mice, and these inductive events were significantly decreased in cells from IL-13Rα2 null animals (Figure 2D). These effects were at least partially Chi3l1/YKL-40/BRP-39 specific because the related GH 18 moiety, acidic mammalian chitinase (AMCase), did not signal in a similar manner via IL-13Rα2 (Figure S2C). Interestingly, Chi3l1/YKL-40/BRP-39 signaling was also significantly decreased in experiments in which soluble IL-13Rα2 was added to the cell culture system (Figures 2E and S2D). These studies demonstrate that Chi3l1/YKL-40/BRP-39 activates ERK and AKT signaling and induces β-catenin nuclear translocation and c-*fos* accumulation via an IL-13Rα2-dependent mechanism(s) in mice and humans and that this signaling is regulated by sIL-13Rα2.

Given that the intracellular domain of IL-13Rα2 only contains a 17-amino-acid structure that lacks protein binding motifs, studies were undertaken to define the role of this domain in Chi3l1/YKL-30/BRP-39 signaling events. To address this question, we compared the signaling induced by Chi3l1/YKL-40/BRP-39 in macrophages from IL-13Rα2 null mice that were transfected with WT (full-length) IL-13Rα2 constructs or truncated constructs that lacked the intracellular domain. These studies demonstrate that the intracellular domain of IL-13Rα2 is not required for Chi3l1 activation of MAPK or AKT (Figure 2F). Interestingly, this segment was required for Chi3l1 activation of the Wnt/β-catenin pathway (Figure S2E). These studies demonstrate that the intracellular domain of IL-13Rα2 has different roles in Chi3l1-induced MAPK and AKT versus Wnt/β-catenin signaling.

### Interactions of IL-13, IL-13Rα2, and Chi3l1/YKL-40/BRP-39

**Figure 3:**
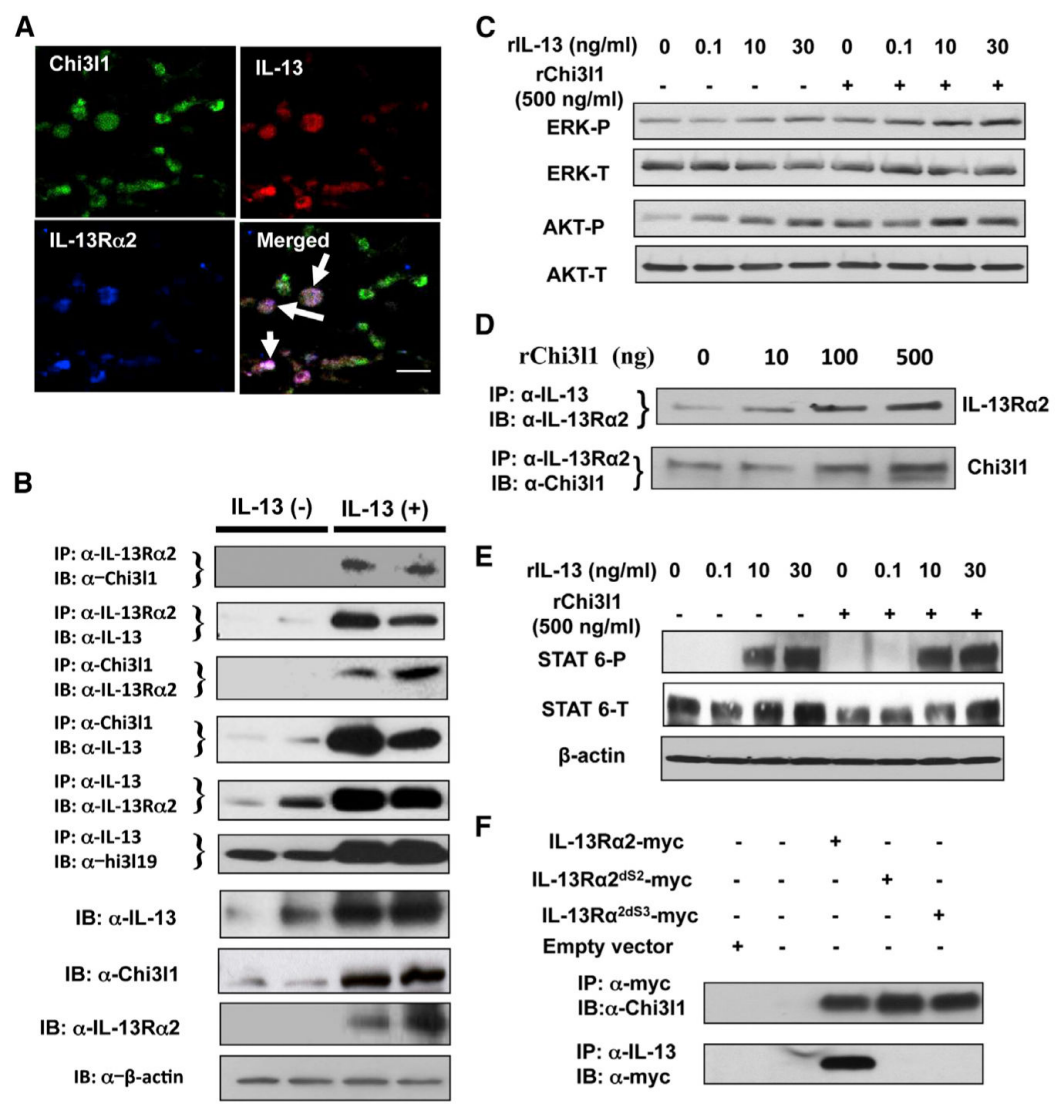
Interactions of IL-13, IL-13Rα2, and Chi3l1/YKL-40/BRP-39 (A) Triple-label IHC was used to detect the colocalization of IL-13, IL-13Rα2, and Chi3l1/BRP-39 in lungs from IL-13 Tg mice using antibodies to Chi3l1/BRP-39, IL-13Rα2, and IL-13. Arrows highlight some of the colocalized cells. (B) Lung lysates were prepared from WT and IL-13 transgenic mice and immunoprecipitated (IP) with anti-Chi3l1, anti-iL-13Rα2, or anti-iL-13, and the precipitates were evaluated using immunoblot (IB) analysis as noted. (C) THP-1 cells were incubated with recombinant (r) IL-13 or Chi3l1/YKL-40 (rChi3l1) alone and in combination as noted. Western blot evaluations were used to evaluate ERK1/2 phosphorylation, total ERK1/2, AKT phosphorylation, and total AKT. (D) Peritoneal macrophages from Chi3l1^−/−^ mice were incubated overnight with rIL-13 (10 ng/ml), cell lysates were prepared, rChi3l1 was added at the noted doses, and immunoprecipitation and immunoblot analysis was undertaken with anti-iL-13 or anti-iL-13Rα2 antibodies as noted. (E) THP-1 cells were incubated with rIL-13 and rChi3l1 as indicated and western blot evaluations were used to evaluate STAT6 phosphorylation (STAT6-P) and total STAT 6 (STAT6-T). (F) Peritoneal macrophages were isolated from IL-13Rα2^−/−^ mice and transfected with empty vector, full-length IL-13Rα2 (IL-13Rα2-myc), or deletion mutants lacking IL-13 binding site 2 (IL-13Rα2^dS2^-myc) or site 3 (IL-13Rα2^dS3^-myc). Cell lysates were prepared, incubated with rChi3l1 (500 ng) and rIL-13 (10 ng) overnight, and evaluated using IP and IB with anti-myc, anti-iL-13, or anti-Chi3l1 as noted.

Because IL-13 also binds to IL-13Rα2, we used coIP, signaling, and Biacore experiments to define the interactions of IL-13, Chi3l1/YKL-40/BRP-39, and IL-13Rα2. These studies demonstrated that IL-13 and Chi3l1/YKL-40/BRP-39 do not bind to one another (data not shown). They did, however, demonstrate that Chi3l1, IL-13Rα2, and IL-13 colocalize in tissue sections (Figure 3A) and participate in a multimeric complex, because the immunoprecipitation of any one of the three always immunoprecipitated the other two (Figure 3B). In keeping with this finding, studies that demonstrated that IL-13 can activate MAPK (Cho et al., 2006; Lee et al., 2006) and studies that demonstrated that IL-13 stimulates heparin binding-epidermal growth factor (HB-EGF) via an IL-13Rα2-dependent mechanism (Allahverdian et al., 2008) demonstrated that IL-13 also activates macrophage MAPK and AKT via IL-13Rα2- and Chi3l1-dependent pathways (Figure S3A and S3B). IL-13 did not compete with Chi3l1/YKL-40/BRP-39 for IL-13Rα2 binding or signaling (Figure 3C). Similarly, Chi3l1/YKL-40/BRP-39 did not compete with IL-13 for IL-13Rα2 binding or signaling (Figure 3D). IL-13 was also a powerful activator of STAT6, Chi3l1/YKL-40 did not activate STAT6, Chi3l1 did not inhibit IL-13-induced phosphorylation of STAT6, and IL-13 did not inhibit Chi3l1/YKL-40 activation of MAPK, AKT, or Wnt/β-catenin signaling (Figure 3E). Structure-function evaluations also demonstrated that deletion of amino acids corresponding to site II (IL-13Rα2^dS2^) and site III (IL-13Rα2^dS3^) of IL-13Rα2, the reported IL-13 binding sites in IL-13Rα2 (Lupardus et al., 2010), abrogates IL-13 binding without abrogating Ch13l1 binding (Figure 3F). These studies demonstrate that IL-13, Chi3l1/YKL-40, and IL-13Rα2 participate in a multimeric complex. They also demonstrate that Chi3l1/YKL-40 and IL-13 do not compete with one another for IL-13Rα2 binding, do not abrogate each other’s signaling, and do not bind to identical locations on IL-13Rα2.

### Role of IL-13Rα2 in Oxidant-Induced Cell Death Responses

**Figure 4:**
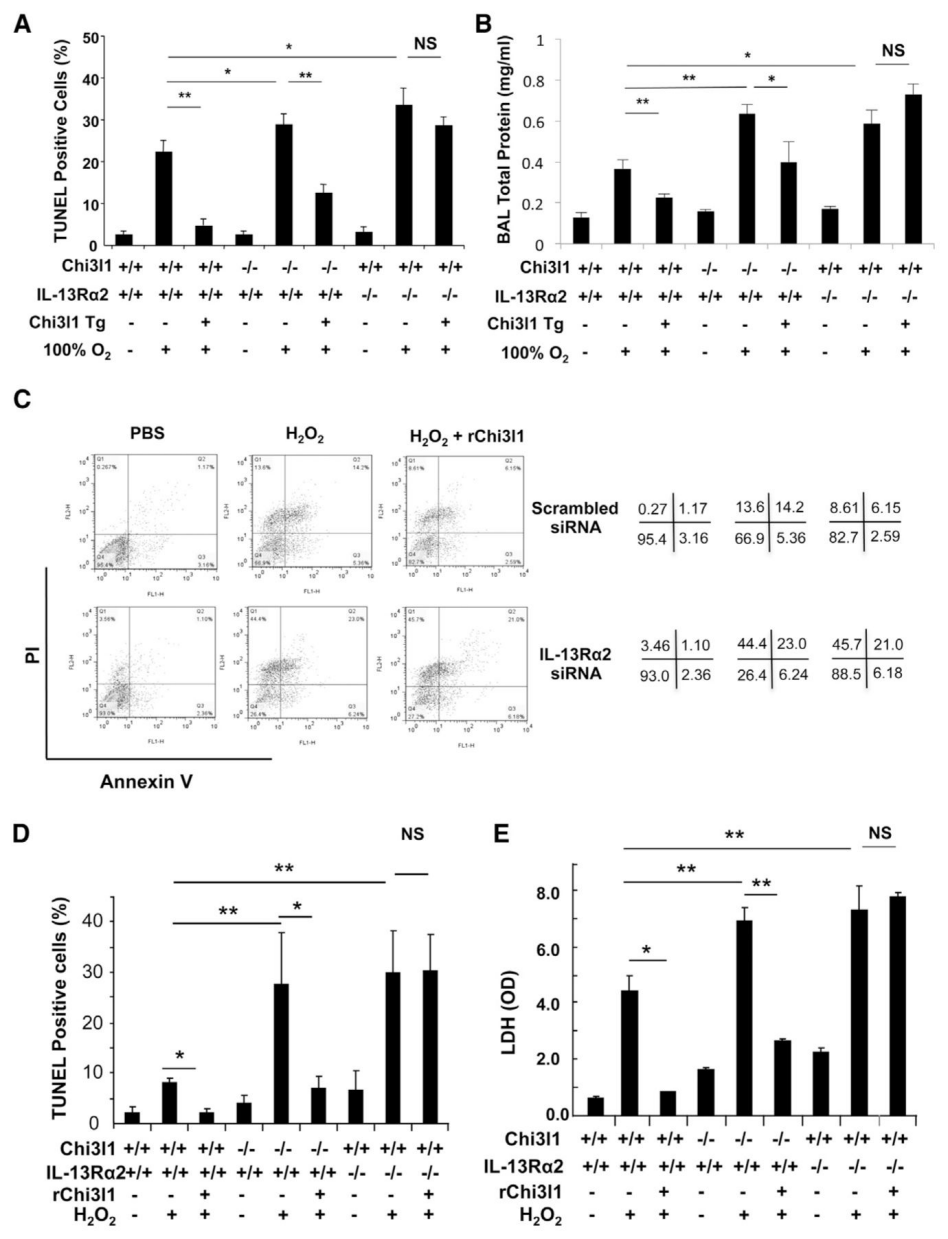
Chi3l1 and IL-13Rα2 Regulation of Cell Death and Acute Oxidant Injury WT (+/+), Chi3l1/BRP-39 null (−/−), IL-13Rα2 null (−/−), Chi3l1/YKL-40 Tg (+), Chi3l1/YKL-40 Tg(+)/BRP-39(−/−), and Chi3l1/YKL-40 Tg(+)/IL-13Rα2(−/−) mice were exposed to 100% O_2_ or room air (100% O_2_ −) for 48 hr. (A) TUNEL staining of lung sections from those mice. (B) BAL fluid total protein. (C) Airway epithelial cells (1HAEo) were exposed for 6 hr to H_2_O_2_ (800 μM). Cells were used that had been treated with IL-13Rα2-specific siRNA or scrambled controls and incubated in the presence and absence of recombinant(r) Chi3l1. Annexin V and propidium iodide staining was evaluated by FACS analysis. (D and E) Peritoneal macrophages from WT (+/+), Chi3l1 null (−/−), and IL-13Rα2 null (−/−) mice were incubated with H_2_O_2_ (H_2_O_2_ +) or its vehicle control (H_2_O_2_ −) in the presence and absence of rChi3l1/BRP-39, and TUNEL staining (D) and LDH release (E) were evaluated. Values in (A), (B), (D), and (E) represent the means ± SEM of evaluations of a minimum of four mice. (C) is representative of at least three similar evaluations. *p < 0.05, **p < 0.01. NS, not significant. See also Figure S4.

Previous studies from our laboratory demonstrated that Chi3l1/YKL-40/BRP-39 inhibits oxidant-induced lung injury and epithelial cell apoptosis (Sohn et al., 2010). To determine if IL-13Rα2 plays a role in these responses, we compared the epithelial cell death and pulmonary injury responses in WT, IL-13Rα2 null, YKL-40 Tg, and YKL-40 Tg/IL-13Rα2^−/−^ mice exposed to room air or 100% O_2_ for 48 hr. Hyperoxia induced epithelial apoptosis/DNA injury, alveolar-capillary protein leak, and caspase-3 and caspase-8 activation in lungs from WT mice (Figures 4A, 4B, and S4A; data not shown). Hyperoxia-induced epithelial TUNEL responses and alveolar protein leak were exaggerated in lungs from Chi3l1/BRP-39 null mice and a phenocopy was seen in IL-13Rα2^−/−^ animals (Figures 4A and 4B). Transgenic YKL-40 ameliorated the hyperoxia-induced responses in WT mice and rescued the exaggerated responses in Chi3l1 null animals (Figures 4A and 4B). Importantly, the protective effects of YKL-40 were significantly decreased in Tg mice that lacked IL-13Rα2 (Figures 4A and 4B). In accord with these in vivo findings, epithelial cells treated with H_2_O_2_ in vitro manifest increased levels of apoptosis and necrosis and these responses were exaggerated in cells that lacked Chi3l1/YKL-40 or IL-13Rα2 (Figures 4C and S4B). The addition of rChi3l1/YKL-40 to these cultures markedly decreased the cell death responses in WT cells and rescued the exaggerated responses in Chi3l1/BRP-39 null mice but did not cause comparable cytoprotection in cells that lacked IL-13Rα2 (Figures 4C and S4B). These responses were not H_2_O_2_ or lung epithelial cell specific because similar responses were seen with FasL-treated lung epithelial cells and H_2_O_2_-treated kidney epithelial cells (Figures S4C and S4D). They were also at least partially mediated by the ability of Chi3l1/YKL-40/BRP-39 to activate AKT, because selective AKT inhibitors abrogated the antiapoptotic effects of Chi3l1/YKL-40/BRP-39 (Figure S4E; data not shown). Similarly, exposure of murine peritoneal macrophages from WT mice to H_2_O_2_ induced TUNEL staining and lactate dehydrogenase (LDH) release, and these responses were further exaggerated in cells from Chi3l1/BRP-39 null animals (Figures 4D and 4E). A phenocopy was seen in cells from IL-13Rα2 null mice, which also manifest exaggerated levels of TUNEL staining and LDH release after H_2_O_2_ treatment (Figures 4D and 4E). Once again, the TUNEL staining and LDH release from macrophages from WT and Chi3l1/BRP-39 null mice were significantly ameliorated by treatment with rChi3l1/BRP-39. In contrast, rChi3l1/YKL-40 did not cause a similar degree of cytoprotection in cells that lacked IL-13Rα2 (Figures 4D and 4E). When viewed in combination, these studies demonstrate that oxidant-induced injury and cell death responses are similarly increased in vivo and in vitro in lungs, epithelial cells, and macrophages from Chi3l1/BRP-39 null and IL-13Rα2 null mice. They also demonstrate that transgenic and recombinant Chi3l1/YKL-40 rescue these exaggerated responses via IL-13Rα2-dependent mechanisms.

### Role of IL-13Rα2 in Pyroptosis

**Figure 5:**
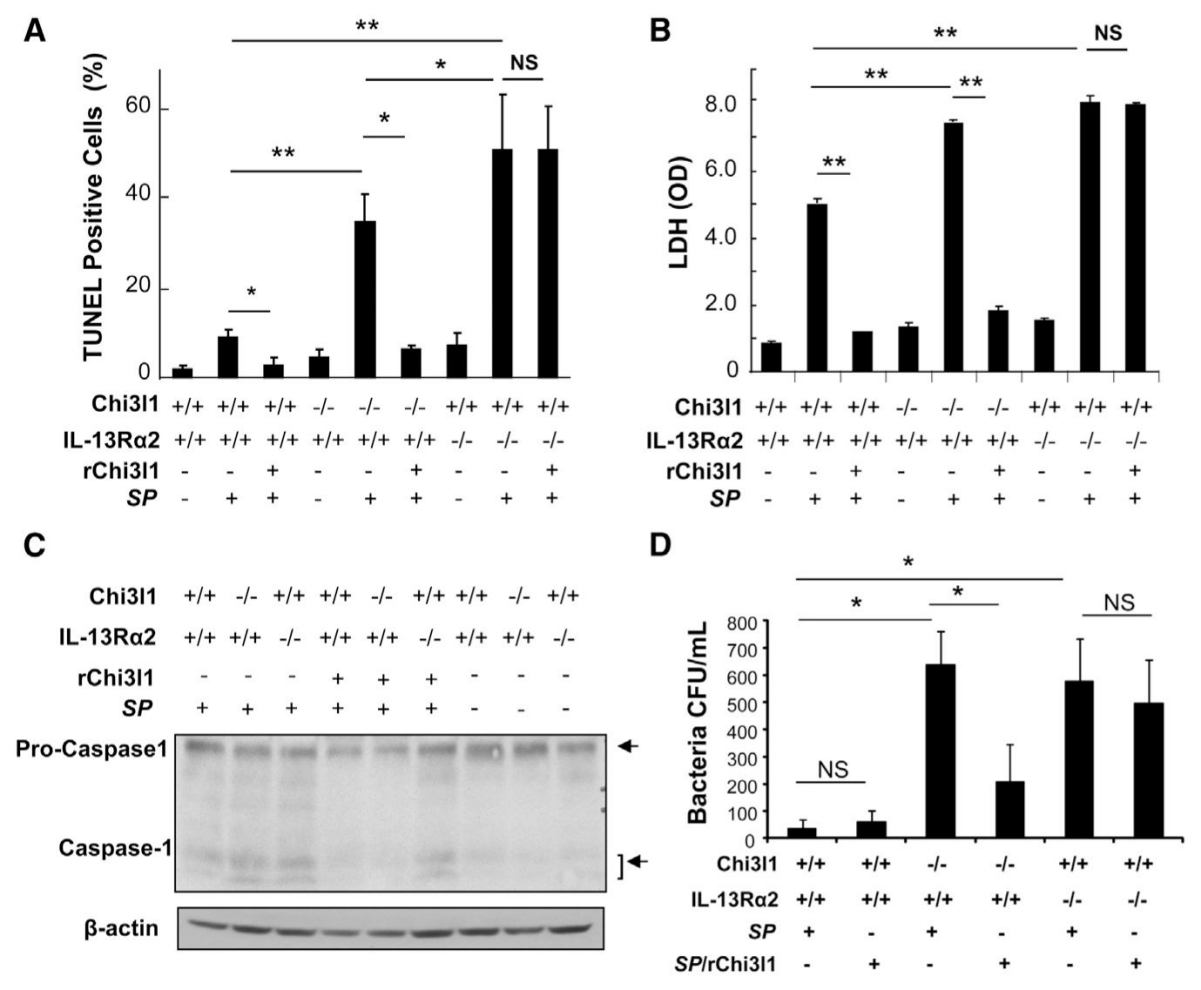
Chi3l1 and IL-13Rα2 Regulation of *SP*-Induced Responses (A and B) Peritoneal macrophages from WT (+/+), Chi3l1 null (−/−), and IL-13Rα2 null (−/−) mice were incubated with *SP* (*SP*+) or its vehicle control (*SP*−) in the presence and absence of rChi3l1/BRP-39, and TUNEL staining (A) and LDH release (B) were assessed. (C) Western blotting was also used to evaluate cell lysate caspase-1 activation. (D) Peritoneal macrophages from WT (+/+), Chi3l1 null (−/−), and IL-13Rα2 null (−/−) mice were also incubated with *SP* (*SP*+) or *SP* preincubated for 60 min without antibiotics with rChi3l1/BRP-39 at a 10:1 *SP*-to-cell ratio (*SP*/rChi3l1). They were then incubated with gentamicin to kill extracellular bacteria and the viable bacteria in the cell lysates were assessed 6 hr later. Values in (A), (B), and (D) are the means ± SEM of triplicate measurements and are representative of a minimum of three similar evaluations; (C) is representative of three similar evaluations. *p < 0.05, **p < 0.01. NS, not significant.

Because previous studies from our laboratory demonstrated that Chi3l1/YKL-40/BRP-39 controls *Streptococcus pneumoniae* (*SP*)-induced macrophage pyroptosis, *SP*-induced cell survival, LDH release, and caspase-1 activation were evaluated in cells from WT, Chi3l1/BRP-39 null, and IL-13Rα2 null mice. Incubation with *SP* increased TUNEL staining and LDH release from cells from WT mice, and this response was further exaggerated in cells from Chi3l1/BRP-39 null animals (Figures 5A and 5B). These responses in cells from WT mice were not associated with significant increases in caspase-1 activity (Figure 5C). However, increased caspase-1 was seen in *SP*-infected cells from Chi3l1/BRP-39 null mice (Figure 5C). A phenocopy was seen in cells from IL-13Rα2 null mice, which also manifest exaggerated levels of TUNEL staining, LDH release, and caspase-1 activation after *SP* infection (Figures 5A–5C). Importantly, the exaggerated TU-NEL staining, LDH release, and caspase-1 activation in Chi3l1/BRP-39 null mice were significantly rescued by treatment with rChi3l1/BRP-39. In contrast, rBRP-39/YKL-40 did not comparably rescue the exaggerated cell death phenotype and cas-pase-1 activation in IL-13Rα2 null cells (Figures 5A–5C). In accord with these studies, the defects in bacterial killing that were seen in macrophages from Chi3l1/BRP-39 null mice (Dela Cruz et al., 2012) were phenocopied in macrophages from IL-13Rα2 null mice (Figure 5D), and the former was rescued with rChi3l1 while the latter was not significantly altered by this recombinant protein (Figure 5D). When viewed in combination, these studies demonstrate that *SP*-induced pyroptosis is increased in a similar manner in macrophages from Chi3l1/BRP-39 null and IL-13Rα2 null mice and that rChi3l1/BRP-39 rescues this exaggerated phenotype via an IL-13Rα2-dependent mechanism.

### Role of IL-13Rα2 in Inflammasome Activation

**Figure 6:**
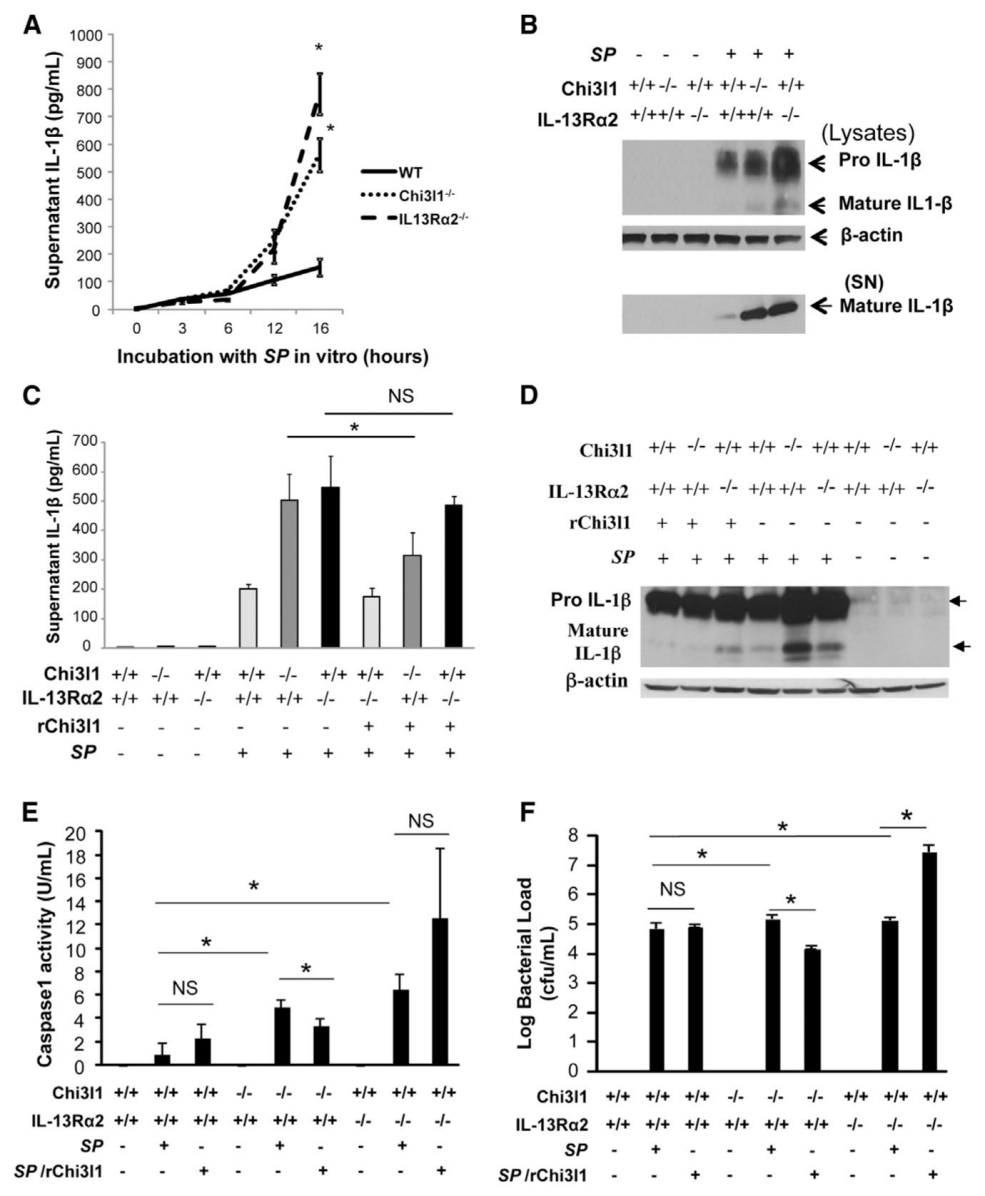
Chi3l1 and IL-13Rα2 Regulation of *SP*-Induced Inflammasome Activation Peritoneal macrophages were obtained from WT mice, Chi3l1/BRP-39 null (−/−) mice, and IL-13Rα2 null (−/−) mice and incubated with *SP*. (A) The kinetics of IL-1β production were evaluated by ELISA. (B) IL-1β maturation and release into the cell supernatant (SN) was evaluated by western blotting. (C–F) The ability of rmChi3l1/BRP-39 to modulate IL-1β production (C) and maturation (D) caspase-1 activity measured by ELISA (E) and bacterial load were also evaluated. Values in (A), (C), (E), and (F) are the means ± SEM of triplicate measurements and are representative of a minimum of three similar evaluations. (B) and (D) are representative of three evaluations. *p < 0.05. NS, not significant.

Studies were also undertaken to define the role(s) of IL-13Rα2 in the regulation of inflammasome activation by Chi3l1/YKL-40/BRP-39. In these experiments, incubation of macrophages from WT mice with *SP* stimulated the production of IL-1β and the accumulation of IL-1β mRNA (Figure S5A). Under these circumstances, a significant increase in pro-IL-1β and a more modest increase in the levels of mature IL-1β could also be appreciated in cells and supernatants from these cultures (Figures 6A–6D). Both of these responses were exaggerated in cells from Chi3l1/BRP-39 null mice (Figures 6A–6D). Enhanced levels of pro-IL-1β were also seen in cells from *SP*-treated IL-13Rα2 null mice (Figures 6A–6D). In keeping with the increased levels of caspase-1 activity in these cells, increased levels of cell-associated and supernatant mature IL-1β were also seen in cultures of *SP*-treated IL-13Rα2 null cells (Figures 6B and 6D). Importantly the exaggerated mature IL-1β responses in Chi3l1/BRP-39 null mice were ameliorated by treatment with rChi3l1/BRP-39. In contrast, rChi3l1/BRP-39 did not rescue the exaggerated inflammasome activation in IL-13Rα2 null cells (Figures 6C and 6D). These studies demonstrate that Chi3l1/YKL-40/BRP-39 inhibits *SP*-induced inflammasome activation via an IL-13Rα2-dependent mechanism.

### Role of IL-13Rα2 in SP infection In Vivo

Previous studies from our laboratory demonstrated that Chi3l1/BRP-39 plays a critical role in antipneumococcal responses where it augments bacterial killing by inhibiting macrophage pyroptosis and inhibits inflammasome activation (Dela Cruz et al., 2012). In keeping with the in vitro observations noted above, studies were undertaken to define the roles of IL-13Rα2 in in vivo anti-*SP* responses. *SP* infection caused a brisk neutrophil-rich inflammatory response that was associated with significant induction of BAL IL-1β (Figures S5B and S5C). These responses and pyroptosis-associated caspase-1 activation were exaggerated and bacterial clearance was decreased in Chi3l1/BRP-39 null mice (Figures 6E and 6F). The *SP*-induced responses in IL-13Rα2 null mice were very similar to those in the Chi3l1/BRP-39 null mice with enhanced BAL and tissue inflammation, IL-1β production, caspase-1 activity, and bacterial accumulation (Figures S5B, S5C, 6E, and 6F). Importantly, rChi3l1/YKL-40 successfully ameliorated the exaggerated responses in Chi3l1/BRP-39 null mice but did not have similar corrective effects in mice that lacked IL-13Rα2 (Figures S5B, S5C, 6E, and 6F). These studies demonstrate that IL-13Rα2 plays an important role in Chi3l1/YKL-40/BRP-39 regulation of pulmonary antibacterial responses in vivo.

### Role of IL-13Rα2 in Chi3l1/YKL-40 Stimulation of Melanoma Metastasis and TGF-β1 Production In Vivo

**Figure 7:**
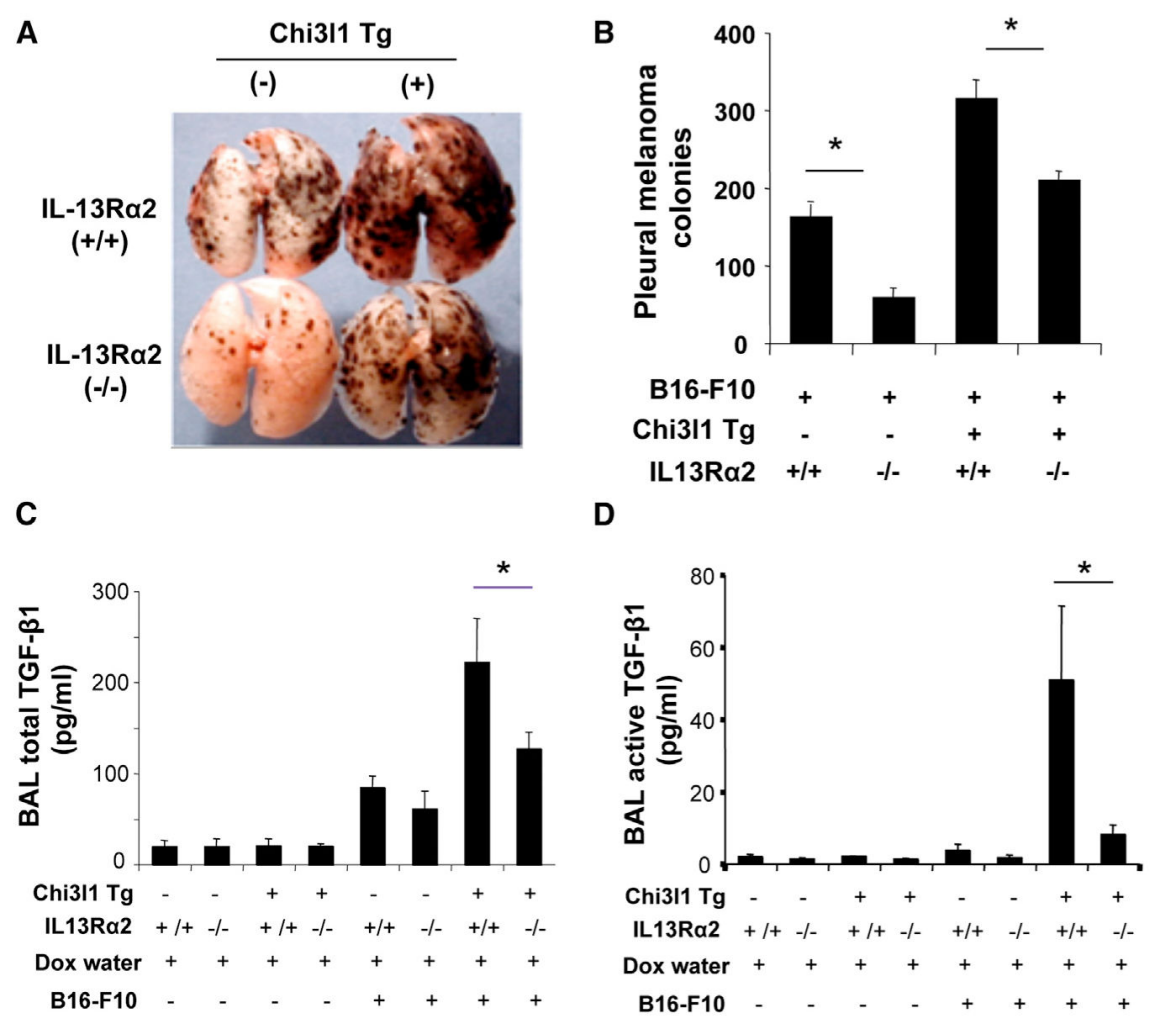
Chi3l1 and IL-13Rα2 in Melanoma Metastasis and TGF-β1 Production In Vivo WT mice, IL-13Rα2 null (−/−) mice, Chi3l1/YKL-40 Tg mice (Tg+), and Chi3l1/YKL-40 Tg/IL-13Rα2 null mice received B16-F10 melanoma cells by tail-vein injection. (A) Fourteen days later, melanoma metastasis was visually assessed. (B) Quantification of pleural melanoma colonies. (C) ELISA evaluation of total BAL TGF-β1. (D) ELISA evaluation of activated TGF-β1. (A) is representative of three similar evaluations. Values in (B)–(D) are the means ± SEM of triplicate measurements and are representative of a minimum of three similar evaluations. *p < 0.05.

Recent studies have demonstrated that pulmonary melanoma metastasis is mediated via an IL-13Rα2-dependent mechanism that requires the production of TGF-β1 (Fichtner-Feigl et al., 2008b; Strober et al., 2009). As a consequence, studies were undertaken to determine if Chi3l1/YKL-40/BRP-39 plays a role in this response and if it does so via IL-13Rα2. To accomplish this, we compared the B16-F10 melanoma cell-induced metastasis and TGF-β1 elaboration in WT mice, YKL-40 Tg mice, and IL-13Rα2 null mice. Melanoma cell administration caused impressive levels of metastasis in lungs from WT mice, and this metastatic response was markedly increased in Tg mice in which YKL-40 was selectively targeted to the lung (Figures 7A and 7B). In accord with the studies of Strober et al. (Fichtner-Feigl et al., 2008b; Strober et al., 2009), these meta-static responses were associated with modest increases in the levels of TGF-β1 in WT mice and significantly enhanced levels of total and activated TGF-β1 in melanoma-challenged YKL-40 Tg animals (Figures 7C and 7D). Importantly, the metastatic responses in the WT and Tg mice and the levels of TGF-β1 production were both significantly decreased in mice with null mutations of IL-13Rα2 (Figures 7A–7D). When viewed in combination, these studies demonstrate that endogenous Chi3l1/BRP-39 and transgenic Chi3l1/YKL-40 regulate pulmonary melanoma metastasis and the TGF-β1 elaboration that underlies these responses. They also demonstrate that the effects of Chi3l1/YKL-40/BRP-39 in this setting are mediated, at least in part, by IL-13Rα2.

## DISCUSSION

IL-13Rα2 was described as a high-affinity receptor for IL-13 that is distinct from the IL-13Rα1-IL-4Rα receptor dimer that IL-13 shares with IL-4 (Lupardus et al., 2010; Strober et al., 2009). It was initially believed to be a decoy receptor because it only contains a 17-amino-acid cytoplasmic domain that lacks a conserved box 1 region that plays a critical role in signal transduction (Konstantinidis et al., 2008) and early studies highlighted its ability to diminish IL-13 responses (Chiaramonte et al., 2003; Lupardus et al., 2010; Wood et al., 2003). However, more recent studies have demonstrated that IL-13 also signals and regulates a variety of cellular and tissue responses via IL-13Rα2 (Daines et al., 2006; Fichtner-Feigl et al., 2006, 2007, 2008a, 2008b; Strober et al., 2009; Yang et al., 2010, 2011). The explanation for these different points of view has not been defined. The present studies, however, provide insights into mechanisms that may contribute to these disparate findings because they demonstrate that IL-13 is not the only ligand for IL-13Rα2. Specifically, they characterize the first receptor for any GH 18 moiety by demonstrating that the chitinase-like protein (CLP) Chi3l1 binds to, signals, and regulates oxidant injury, apoptosis, pyroptosis, inflammasome activation, pathogen responses, melanoma metastasis, and TGF-β_1_ via IL-13Rα2.

Endogenous lectins such as C-type lectins, siglecs, and galectins bind N- and O-linked glycans, resulting in regulatory signals that control immune cell homeostasis and integrate circuits that amplify or silence immune responses (Rabinovich and Croci, 2012). These lectins recognize complex glycan determinants with relatively high affinity, often in the micromolar range (Rabinovich and Croci, 2012; Sulak et al., 2009). CLPs are also lectins and are frequently referred to as chilectins. In keeping with other lectin-glycan interactions, our studies demonstrate that Chi3l1 binds to the glycopeptide IL-13Rα2 (Kioi et al., 2006) with high affinity. They also demonstrate that this interaction is dependent on the CD and chitin binding motif of Chi3l1 and the extracellular domain of IL-13Rα2 but did not require IL-13Rα2 N-glycosylation. When combined with our demonstration that Chi3l1 signals and regulates cellular and tissue responses, these findings allow for the exciting hypothesis that IL-13Rα2 is a receptor for Chi3l1, putting the Chi3l1-IL-13Rα2 complex at the interface of glycobiology and protein biology (Coffman, 2008). It is important to point out, however, that our studies do not demonstrate that IL-13Rα2 is the only receptor for Chi3l1/YKL-40//BRP-39. In fact, our studies suggest that other receptors may exist because, in some of our experimental systems, the elimination of IL-13Rα2 only partially abrogated the specific Chi3l1/YKL-40/BRP-39 effector response.

Studies were also undertaken to define the interactions of IL-13, IL-13Rα2, and Chi3l1/YKL-40/BRP-39. These studies demonstrate that these moieties exist in tissues and fluids in a multimeric complex. Our studies do not address the details of the Chi3l1-IL-13Rα2-IL-13 complex. However, modeling of other lectin-glycan interactions has revealed two- and three-dimensional arrangements of multivalent lectins and glycans in “lattices” that serve as scaffolds that organize plasma membrane domains and modulate the signaling thresholds of relevant surface glycoproteins and receptors (Rabinovich and Croci, 2012). Thus, it is possible that Chi3l1, IL-13Rα2, and IL-13 are part of a large multimeric complex or “chitosome” that could include other glycoproteins and lectins. Additional investigation will be required to evaluate the nature of the IL-13Rα2-Chi3l1 complex and its relationship to IL-13 and other Chi3l1 receptors.

Using concentrations of Chi3l1 that can be seen in the circulation of healthy individuals and in patients with disease, these studies demonstrated that Chi3l1 can activate MAPK and AKT cellular signaling pathways (Areshkov et al., 2012; Chen et al., 2011a; Eurich et al., 2009; Kim et al., 2012; Shao et al., 2009). Our studies added to these observations by demonstrating that Chi3l1 activates the Wnt/β-catenin signaling and by demonstrating that IL-13Rα2 is required for the optimal activation of each of these signaling pathways. These findings are in keeping with the reported ability of IL-13 to activate MAPKs, Wnt/β-catenin, and AKT (Moriya et al., 2011; Ooi et al., 2012; Wang et al., 2008) and the ability of IL-13 to stimulate epithelial HB-EGF via IL-13Rα2 (Allahverdian et al., 2008). Lastly, our studies demonstrate that the antiapoptotic effects of Chi3l1/YKL-40/BRP-39 are mediated, at least in part, by its ability to activate AKT. When viewed in combination, it is tempting to speculate that these signaling events, in addition to their contribution to the anti-apoptotic effects of Chi3l1, also contribute to its inflammatory, angiogenic, neoplastic, and other effector responses (Chen et al., 2011b; Coffman, 2008; Eurich et al., 2009; Faibish et al., 2011; Kawada et al., 2012; Lee et al., 2009; Shao et al., 2009).

To define the role(s) of the intracellular domain of IL-13Rα2 in the signaling events that were noted, we compared the effects of Chi3l1 in cells from IL-13Rα2^−/−^ mice that were transfected with constructs that encoded full-length IL-13Rα2, constructs that lacked the intracellular domain of IL-13Rα2, and controls. These studies demonstrated that the MAPK and AKT activation events did not require the intracellular domain of the receptor. In contrast, the intracellular domain played a critical role in the activation of the Wnt/β-catenin/AP-1 pathway. The latter findings are in full accord with reports by Fichtner and Feigl (Fichtner-Feigl et al., 2006). When viewed in combination, these findings highlight the different roles of the intracellular domain of IL-13Rα2 in Chi3l1-induced cell signaling. They also raise the interesting question, how does Chi3l1 activate ERK and AKT without an intra-cellular domain? Preliminary studies in our laboratory support the possibility that a coreceptor may be involved. However, additional investigation will be required to fully address this possibility.

Chi3l1/YKL-40/BRP-39 may play a particularly important role in cancer (Coffman, 2008; Goel et al., 2007; Lee et al., 2011). Recent studies by the Strober group also highlighted the importance of IL-13Rα2 in the progression of malignant melanoma, where its activation by IL-13 caused TGF-β1 elaboration, which inhibited tumor immune surveillance and favored tumor growth (Fichtner-Feigl et al., 2008b; Strober et al., 2009). Previous studies from our laboratory demonstrated that IL-13 stimulates TGF-β1 via a Chi3l1/BRP-39-dependent mechanism (Lee et al., 2009). The present studies add to these observations by demonstrating that Tg Chi3l1/YKL-40 stimulates TGF-β1 in lungs with metastatic melanoma via an IL-13Rα2-dependent mechanism. Interestingly, they also demonstrated that IL-13Rα2 plays a particularly important role in the production of bioactive TGF-β1 in this setting. Thus, in accord with the findings by Strober et al. and prior studies that demonstrate that the levels of circulating Chi3l1/YKL-40 are increased in patients with advanced melanoma (Schmidt et al., 2006), an IL-13-Chi3l1/YKL-40-IL-13Rα2-TGF-β1 axis appears to play a critical role in the progression of malignant melanoma. These studies also suggest that IL-13Rα2-dependent mechanisms may play an important role in TGF-β1 activation.

In keeping with the retention of CLP over species and evolutionary time, recent studies have demonstrated that Chi3L1 plays essential roles in pathogen clearance and the generation of host tolerance (Dela Cruz et al., 2012). In the setting of pneumococcal lung infection, Chi3l1 augments bacterial killing and clearance by controlling pyroptosis (Dela Cruz et al., 2012). This prevents the bacteria from killing the macrophage before the macrophage can kill the bacteria (Dela Cruz et al., 2012). Simultaneously, Chi3l1 diminishes innocent-bystander tissue injury by controlling innate immune inflammasome and purinergic pathway activation (Dela Cruz et al., 2012) and decreases tissue oxidant injury (Sohn et al., 2010). To define the roles of IL-13Rα2 in these events, we characterized these responses in WT mice and Chi3l1/BRP-39 null mice and evaluated the ability of Tg Chi3l1/YKL-40 to rescue these responses in mice with WT and null IL-13Rα2 loci. These studies demonstrate that, in the absence of Chi3l1/BRP-39, pneumococcus causes exaggerated macrophage cell death, inflammation, and tissue injury and decreased bacterial clearance and that each of these responses was significantly ameliorated by Tg Chi3l1/YKL-40. They also demonstrate that, in the absence of Chi3l1/BRP-39, oxidants caused exaggerated cellular apoptosis, which was rescued by Chi3l1/YKL-40. Importantly, qualitatively similar responses were seen in IL-13Rα2 null animals and cells exposed to pneumococcus or H_2_O_2_. However, the ability of Chi3l1/YKL-40/BRP-39 to rescue the phenotypes in IL-13Rα2 null mice and or cells was decreased compared to the effects of Chi3l1/YKL-40 in mice and cells that lacked Chi3l1/BRP-39. This demonstrates that Chi3l1 controls pyroptosis, apoptosis, inflammasome activation, and antipneumococcal responses by binding to and activating IL-13Rα2. This further reinforces the concept that IL-13Rα2 is a receptor for Chi3l1 and more than just a “decoy” receptor for IL-13.

## EXPERIMENTAL PROCEDURES

### Mice

C57BL/6 mice (the Jackson Laboratory) were bred at Yale. Chi3l1/BRP-39 null mutant mice (Chi3l1^−/−^) and YKL-40 and IL-13 Tg mice were generated and characterized in our laboratory as previously described (Lee et al., 2009; Zhu et al., 1999). IL-13Rα2 null mice (IL-13Rα2^−/−^) were purchased from The Jackson Laboratory and backcrossed to C57BL/6 backgrounds. All murine procedures were approved by the Institutional Animal Care and Use Committee at Yale University.

### Yeast Two-Hybrid Screening

The full-length murine Chi3l1 gene was amplified by PCR from mouse lung complementary DNA (cDNA) using the following primers: forward, 5′-CCCCGGGCTGCAGGGATCCGG CAGAGAGAAGCCATC-3′; reverse, 5′-CATATGGGAAAGGTCGACCTAAGCCAG GGCATCCTT-3′). The Chi3l1 DNA was cloned into the yeast two-hybrid BD vector at the BamHI and *Sal1* sites. The Matchmaker System 3 two-hybrid assay using *S. cerevisiae* (Clontech) was used to detect interactions between Chi3l1 and other cellular proteins. *S. cerevisiae* strain AH109 (Clontech) containing the four reporter genes *ADE2*, *HIS3*, *MEL1*, and *lacZ* was cotransfected with the pGBKT7-Chi3l1 bait plasmid and the mouse lung cDNA library (Clontech) constructed into the vector pAC2 by the lithium acetate method. Additional experimental details are included in the Extended Experimental Procedures.

### Coimmunoprecipitation

Proteins from the lysate, culture supernatant, or BAL fluid were clarified by centrifugation for 10 min at 4°C. Chi3l1/YKL-40 and human IL-13Rα2 were immunoprecipitated with anti-YKL-40 rabbit polyclonal antibody (MedImmune) or anti-hIL-13Rα2 monoclonal antibody (RayBiotech), respectively, using Catch and Release V2.0 (Reversible Immunoprecipitation System, EMD Millipore). The precipitates were subjected to immunoblotting with antibodies against IL-13Rα2 or YKL-40, respectively.

### Double-Label Immunohistochemistry

To localize the expression of BRP-39 and IL-13Rα2, double-label IHC was undertaken with a modification of procedures described previously by our laboratory (Lee et al., 2009). Additional experimental details are included in the Extended Experimental Procedures.

### Expression and Purification of IL-13, IL-13Rα2, and YKL40

Genes encoding residues 21–132 of human IL-13, ectodomain residues 29–331 of human IL-13Rα2 (IL-13Rα2-ECD), or human YKL40 were subcloned into the pAcGP67 vector (BD Biosciences) in frame with the baculovirus gp67 signal sequence and followed by the sequence PHHHHHH. Sf9 insect cells (Invitrogen) were cotransfected with one of the above expression constructs and DiamondBac baculovirus genomic DNA (Sigma-Aldrich) to produce recombinant baculoviruses expressing IL-13 or IL-13Rα2-ECD, or YKL40. Virus stocks were amplified with three sequential infections of Sf9 cells. The three proteins were expressed and purified following the same protocol. *Tni* insect cells (Expression Systems) grown at 27°C were infected at a density of 2 × 10^6^ cells/ml with 1.0% (v/v) of third-passage (P3) recombinant baculovirus stock. After culture in suspension for 96–105 hr at 20°C, the culture media was collected and its pH was adjusted with 10 mM HEPES pH 7.5. The overexpressed protein was purified by nickel-affinity chromatography with a HisTrap HP column (GE Healthcare), followed by size-exclusion chromatography on a Superdex 200 10/300 GL column (GE Healthcare). The size-exclusion buffer was 10 mM HEPES pH 7.5, 50 mM NaCl, and 0.5 mM CaCl_2_. Protein concentrations were measured by UV spectroscopy at 280 nm using a Nanodrop2000 spectrometer (Thermo Scientific).

### Kinetic Binding Analysis by Surface Plasmon Resonance

Surface plasmon resonance experiments were performed with a CM5 sensor chip, at 25°C on a Biacore T100 instrument (GE Healthcare). YKL40 was captured at a low density (2,000–4,000 response units) by direct amine-based coupling. The ethanolamine-blocked surface acted as a reference for the CM5 sensor chip. Dose-response experiments were performed as 3-fold serial dilutions of IL-13Rα2-ECD in running buffer (10 mM HEPES pH 7.4, 150 mM NaCl). The sensor chip was regenerated with 10 mM NaOAc pH 4.0 and 250 mM NaCl. IL-13Rα2/YKL40 binding kinetics were measured during 180 s association and 780 s dissociation phases, with a flow rate of 45 μl/min. Data were analyzed with the Biacore T100 evaluation software version 2.0 with a 1:1 Langmuir binding model. All experiments were performed in triplicate.

### Deletion Mapping

Deletion mutants of Chi3l1/YKL-40 and IL-13Rα2 were generated using PCR amplification using primers listed in Tables S1 and S2. The yeast two-hybrid assay was used to evaluate the interactions of each deletion mutants of Chi3l1 and IL-13Rα2 with full-length IL-13Rα2 and Chi3l1, respectively.

### Nuclear Protein Extraction

The nuclear and cytoplasmic protein fraction from cultured peritoneal macrophage was extracted using NE-PER Nuclear and Cytoplasmic Extraction kit (Thermo Scientific) as per the manufacturer’s instructions.

### Immunoblotting

Protein lysates were prepared from cultured cells or whole lungs using RIPA lysis buffers and subjected to immunoblotting using a modification of procedures described previously by our laboratory (Lee et al., 2009). Additional experimental details are included in the Extended Experimental Procedures.

### RNA Interference Analysis

Human IL-13Rα2 siRNAs (Santa Cruz Biotechnology) were used to knock down IL-13Rα2 according to the protocols provided by the manufacturer. Cells were plated on six-well plates and transfected the next day with IL-13Rα2 or control siRNAs. The cells were harvested at the indicated time points and were subjected to real-time RT-PCR or western blot evaluations.

### Cell Death Evaluations

Cell death and DNA injury were evaluated with TUNEL and FACS analyses of Annexin V and propidium iodide (PI) staining as previously described by our laboratory (Lee et al., 2009). The in vivo cell death response was evaluated after the mice (WT, Chi3l1^−/−^, and IL-13Rα2^−/−^ mice) were exposed to 100% oxygen or room air for up to 3 days as described previously (Sohn et al., 2010). The in vitro evaluations were done under control conditions, after incubation with H_2_O_2_ (J. T. Baker Chemical; 500–800 μg/ml) or after incubation with rFasL (Peprotech). 1HAEo-transformed lung airway epithelial cells were obtained from Dr. D. Greunert (University of California, San Francisco). Peritoneal macrophages and proximal renal tubular epithelial cells were isolated from WT, Chi3l1^−/−^, and/or IL-13Rα2^−/−^ mice in these in vitro cell death evaluations. AKT inhibitor (*Akt-In*) was purchased from EMD Millipore.

### Lactate Dehydrogenase Test

Supernatant LDH was measured using the Cytotoxicity Detection Kit (LDH; Roche Applied Science) as per the manufacturer’s instructions.

### Bacterial Infection

In vivo and in vitro bacterial infection was done as previously described in our laboratory (Dela Cruz et al., 2012).

### Quantification of Caspase-1 Bioactivity

Caspase-1 bioactivity was assessed using the Caspase 1 Colorimetric Assay Kit (Millipore) as per the manufacturer’s instructions.

### Assessment of Melanoma Lung Metastasis

Mouse melanocytes (B16-F10), established from C57BL6/J mouse skin melanoma, were purchased from the American Type Culture Collection (CRL-6475). After culture to confluence in ordinary Dulbecco’s modified Eagle’s medium (DMEM), the cells were delivered to the mice by tail-vein injection (2 × 10^5^ cells/mouse in 200 μl of DMEM). Lung melanoma metastases were quantified by counting the number of colonies (which appear as black dots) on the pleural surface.

### Quantification of TGF-β_1_ and IL-1β

The levels of BAL fluid Th2 cytokines and active and total TGF-β1 (before and after acid activation, respectively) were measured by ELISA using commercial kits (R&D Systems) as directed by the manufacturer.

### Statistical Analysis

Normally distributed data are expressed as mean ± SEM and were assessed for significance by Student’s t test or ANOVA as appropriate. Statistical significance was defined at a p value less than 0.05. All statistical analyses were performed with SPSS version 13.0 (SPSS). Statistical significance was defined at a level of p < 0.05.

## Supplementary Material

Suppl 1
