# Supplementary material for: Chitinase 3-like 1 Regulates Cellular and Tissue Responses via IL-13 Receptor α2
Source: Cell Rep. Author manuscript; Available in PMC 2014 Aug 29. (PMC3988532; doi:10.1016/j.celrep.2013.07.032)

Table S1. Primer Sequences and Locations Used to Generate Chi3l1 Fragments for Deletion Mapping, Related to XXXX

Primer name	Primer Sequence
BD1	ACA TAT ATA CAT ATG ATG GGT GTG AAG GCG CCT CAA
BD2	ATA TAT ATG TCG ACG CTA CGT TGC AGC GAG TGC ATC
BD3	GAG GAC GCT CAT ATG TAC AAA CTG GTC TGC TAC TAC ACC
BD4	ATA TAT ATG TCG ACG TTT GTA TGC AGA GCA GCA CTG
BD5	ATA TAT ATG CAT ATG GAT GAC TTC CAG GGC TCC TTC
BD6	ATA TAT ATG TCG ACG GAA GTC ATC CAG GTC CAG
BD7	ACA TAT ATA CAT ACC TGA AGG ACA GGC AGC TGG CGG
BD8	ATA ATA TAT AGC TGC AGC TAT GGA CTG TGG CTC CGC GGA
BD9	GAG GAC CTG CAT ATG CAT AGA ATC CTC GGC CAG CAG
BD10	CGC TGC AGG TCG ACG CTG CAC CTT GCT TTT GAC GCT

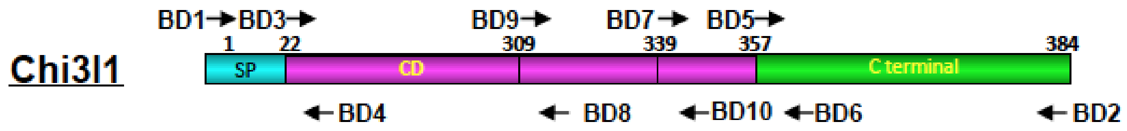

Table S2. Primer Sequences and Locations Used to Generate hIL-13Rα2 Fragments for Deletion Mapping, Related to XXXX

Primer name	Primer Sequence
AD1	AAG GTT TTC CAT ATG GGA GAA ATG GCT TTC GTT TGC
AD2	GTC TCT TGA TAT CTC GAG TCT TCA TGT ATC ACG GAA
AD3	GCA CTG GAT CAT ATG GAC ACC GAG ATA AAA GTT AAC
AD4	TAT GCT ATC TCG AGC TGA AGA TGA AGT ACA GCC AAA
AD5	CAT ACA TCG CAT ATG TTC TGG CTA CCA TTT GGT TTC
AD6	ATA TCA GAC TCG AGC ACG TAG CAA AGT TTT CTT CGA
AD7	TAT ATG ATG CAT ATG CGT AAG CCA AAC ACC TAC CCA
AD8	TAT TAG TAC TCG AGC CAA AAG CAG ACC GGT TAC AAA

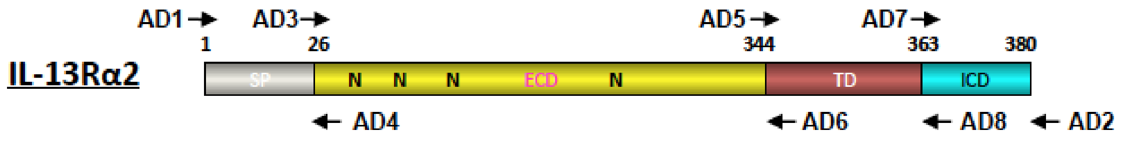

Supplement: Suppl 1 [file NIHMS543175-supplement-Suppl_1.pdf]
